# Evaluation of the Association of Single Nucleotide Polymorphism rs2229238 in Interleukin 6 Receptor Alpha (IL6RA) Gene With the Risk of Preeclampsia

**DOI:** 10.7759/cureus.24788

**Published:** 2022-05-06

**Authors:** Nagarjuna Sivaraj, Vijaya Rachel K, Tarun Kumar Suvvari, Shilaja Prasad, Boppana Sri Harsha, Vineetha Majji, Pradeep Kumar Vegi, Papa Kusuma Bunga

**Affiliations:** 1 Research and Development, Great Eastern Medical School and Hospital, Srikakulam, IND; 2 Biochemistry and Bioinformatics, Gandhi Institute of Technology and Management (Deemed To Be University), Visakhapatnam, IND; 3 Medicine and Surgery, Dr. Nandamuri Taraka Rama Rao (NTR) University of Health Sciences, Vijayawada, IND; 4 Obstetrics and Gynaecology, Great Eastern Medical School and Hospital, Srikakulam, IND; 5 General Medicine, Great Eastern Medical School and Hospital, Srikakulam, IND; 6 Central Research Laboratory, Great Eastern Medical School and Hospital, Srikakulam, IND; 7 Biochemistry, Great Eastern Medical School and Hospital, Srikakulam, IND

**Keywords:** single nucleotide polymorphism, gene, inflammation, interleukins, il6ra, preeclampsia

## Abstract

Introduction

Many studies have gone into single nucleotide polymorphisms (SNPs) in inflammatory-associated genes and preeclampsia risk; still, the findings are inconclusive. The current study aims to evaluate the association of SNP rs2229238 in the interleukin 6 receptor alpha (IL6RA) gene with the risk of preeclampsia.

Methodology

An observational case-control study was conducted and 216 patients were included in this study. Of the patients, 104 were normotensive subjects and 112 were subjects with preeclampsia. Genotyping for SNP rs2229238 was performed by the polymerase chain reaction-restriction fragment length polymorphism (PCR-RFLP) method.

Results

The genotype allocation of the SNP 2229238 C/A polymorphism was not different in preeclampsia subjects (CC: 42%; CA: 42%; AA: 16%) and normotensive pregnant women (CC: 37%; CA: 48%; AA: 15%) (p-value = 0.73). The frequency of the A allele was 34% in preeclampsia subjects and 31% in normotensive pregnancies. There was no significant variation seen in the allele frequencies among cases and the control population.

Conclusion

Our study reported that there is no significant relation between preeclampsia and IL6RA SNP rs2229238. Also, there is no significance in the allele frequencies among both cases and control groups.

## Introduction

Preeclampsia is gestational hypertension associated with proteinuria after 20 weeks of pregnancy or gestational hypertension with or without proteinuria but having significant end-organ dysfunction after 20 weeks of pregnancy [1]. Preeclampsia accounts for 2-8% of pregnancy-related complications globally. In developing countries, 9-26% of maternal deaths were due to preeclampsia and in developed countries, 16% of maternal deaths were due to preeclampsia [1]. The etiopathogenesis of preeclampsia is still unclear and the primary reason for preeclampsia is the dysfunction of maternal systemic endothelial cells [2]. However, the two-stage model of preeclampsia states that “a poorly perfused placenta (Stage 1) produces factor(s) leading to the clinical manifestations of preeclampsia (Stage 2)” [2].

Plasma inflammatory markers such as cytokines, C-reactive protein, and adhesion molecules are responsible for inflammatory changes in preeclampsia [3]. Ischemia and hypoxia in the placental tissue can cause preeclampsia with endothelial dysfunction due to enhancement in the release of a cytokine such as a tumor necrosis factor-α, interleukin 1A (IL-1A), and also interleukin 1β [4].

Many studies have gone into single nucleotide polymorphisms (SNPs) in inflammatory-associated genes and preeclampsia risk; still, the findings cannot draw a better conclusion [5-9]. Initial studies have concentrated on the interleukin-1 receptor 1 (IL-1R1) gene, IL-1A gene, and other associated genes in the Indian population [7-10]. Heterogeneity of preeclampsia and variations in the ethnic groups may affect the results. So, regional studies might help the scientific community to a better conclusion. The main intention of this study is to assess the interleukin 6 receptor alpha (IL6RA) SNPs and their association with the risk of preeclampsia.

## Materials and methods

The interleukin 6 (IL-6) gene polymorphism was prospectively evaluated in 112 preeclampsia subjects presented to the obstetrics and gynecology department. The control group included 104 pregnant women without preeclampsia. Informed consent was obtained from all the participants. Identification and risk evaluation of preeclampsia were adhered to according to the principles of the American College of Obstetricians and Gynecologists (ACOG) 2002 guidelines [11]. Normotensive pregnant women without hypertensive history or any other complications were incorporated as the control population. There were no differences in the ethnicity of controls and cases. By using the autoanalyzer (Roche) of clinical biochemistry, hemoglobin percentage (HB%), total red blood cells (RBCs), total white blood cells (WBCs), and platelet count levels were determined.

Molecular investigation

With the help of the Genomic DNA Purification Kit (GeNei, Peenya, Bangalore), genomic DNA was purified from 3 ml of ethylenediaminetetraacetic acid (EDTA) peripheral blood. IL-6 genotyping for the rs2229238 C/A polymorphism was performed by polymerase chain reaction (PCR). The primers used were forward 5’-TGACAATACCTCGAGCCACA-3’ 5’-ATTTTCCCCTGGCGTAGAAC-3’ reverse primers. During the 30 cycles of DNA amplification, denaturation at 95°C for 30 seconds, annealing at 50.5°C for 30 seconds, and extension at 72°C for 90 seconds were performed, with an extension time of seven minutes at 72°C, followed by a final hold of 4°C.

At 95°C, five minutes were required to complete the initial denaturation step. BtsCI restriction enzyme was used to digest PCR products at 37°C all night. As a result of electrophoresis, PCR digested products were visualized on 2% agarose gel stained with ethidium bromide with GeNei Gel Doc System. It was found that the IL6RA rs2229238 SNP displays the C allele as 341 bp whereas the C allele was separated into 249 bp and 92 bp long. Sanger sequencing of CC genotype was used as a positive control. Restriction fragment length polymorphism (RFLP) patterns were compared between control and preeclampsia cases.

The study was approved by the Institutional Ethics Committee, Great Eastern Medical School and Hospital, Srikakulam, India. The reference number of the ethical approval is 24B/IEC/GEMS&H/2018 and the study was approved on 06/09/2018. The project was self-funded by the investigators.

## Results

This study included 216 subjects, 104 of whom were normotensive and 112 were preeclamptic. There was no significance between age groups among both cases and controls (p-value = 0.9981), but gestational age was significant among both groups (p-value = 0.0000). The anthropometric characteristics and predictable liability of the two groups are shown in Table 1. There is not much difference between blood parameters like hemoglobin (HB), RBC, WBC, and packed cell volume (PCV) among both groups.

**Table 1:** Anthropometric and clinical profile of controls and cases BP: blood pressure; PCV: packed cell volume; OBG: obstetrics and gynecology.

Parameters	Normotensive (n = 104)	Preeclampsia (n = 112)	P-value
Age	23.03 ± 3.15	23.23 ± 3.15	0.9981
Systolic BP	109.81 ± 8.12	145.27 ± 16.87	0.0000001
Diastolic BP	71.25 ± 8.77	95.08 ± 11.15	0.014
Weight (kg)	62.77 ± 9.96	67.83 ± 17.16	0.0000001
Height (cm)	153.02 ± 12.38	149.1 ± 16.39	0.0041
Gestational age	40.176 ± 1.04	35.26 ± 1.76	0.00000013
Blood parameters			
Hemoglobin %	11.22 ± 1.55	10.91 ± 1.76	0.19
Total RBC count	4.19 ± 0.50	4.27 ± 0.57	0.17
Total WBC count	11636 ± 3031.2	11718.75 ± 3271.10	0.43
PCV%	79.41 ± 8.96	77.65 ± 8.42	0.51
Platelet count	1047.8 ± 9807	3877 ± 17423	0.0000001
OBG history			
Primigravidae	54 (52%)	74 (66%)	-
Multigravidae	50 (48%)	38 (33%)	-

Genotype distribution between pregnant women with preeclampsia was CC = 42%, CA = 42%, and AA = 16%, and between pregnant women with normotensive pregnancy was CC = 37%, CA = 48%, and AA = 15% (p-value: 0.73; Table 2). The frequency of the A allele was 34% in preeclampsia subjects as well as 31% during normotensive pregnancies. The allele frequencies between case and control groups did not differ significantly.

**Table 2:** Allele frequencies for controls and cases

Genotype/allele	Normotensive (n = 104)	Preeclampsia (n = 112)	Statistical values
AA	16 (15%)	18 (16%)	χ2 = 0.608, p-value = 0.7378
CA	49 (48%)	47 (42%)	
CC	39 (37%)	47 (42%)	
A	64	76	P-value = 0.55, OR = 1.15 (0.7563-1.767)
C	144	148	

## Discussion

The IL6RA SNP rs2229238 did not show any association with preeclampsia in our study population. We noted that there is no significant variation in the allele frequencies among both cases and control groups. Our study was the first study to evaluate IL6RA polymorphism and its association with preeclampsia among the Indian population.

In a study by Mrema et al., the risk of preeclampsia was predominant among pregnant women in the age group of 35 years or above when compared to young pregnant women [12]. Whereas, in our study, there is no significant association of age group with risk of preeclampsia among both cases and controls. In our study, preeclampsia was more common in primigravida compared to multigravida women, which was similar to a study by Reyes et al. [13].

Fan et al. reported that IL-6 does not appear to be associated with preeclampsia risk by comparison with normotensive pregnant women in the Chinese population, and our study also reported similar results with the risk of preeclampsia [14]. The genotypic summary of the IL-6 gene revealed that there is no contribution in the assorted class of inflammatory-associated diseases like coronary artery disease, coronary heart disease, and Alzheimer's disease [14-17].

T-helper cells can lead to multiple forms of inflammatory cell cytokines, such as interleukins 4, 5, 6, and 10, which might interrupt cellular immunity, increase placental growth, and improve the quality and function of cell inflammatory cytokines [18]. The IL6RA is a specific receptor of IL-6 that could trigger more than three signaling pathways with Btk, Ras/extracellular signal-regulated kinase (ERK), and Janus kinase 2 (JAK2)/signal transducer and activator of transcription 5 (STAT5) [19]. Among them, the Ras/ERK pathway's stimulation can help cell propagation and persistence, in contrast to trophoblastic programmed cell death and aponecrosis, which have been associated with preeclampsia as primary pathological measures [20]. Our study reveals that IL6RA SNP rs2229238 does not trigger any signaling pathways, but other SNPs of IL6RA may trigger signaling pathways and further investigations are needed.

The limitation of our study is that our study is a single hospital study and multicentric global studies are needed for better conclusions. Other genes apart from IL6RA rs2229238 and other SNPs could play a key role in the risk of preeclampsia.

## Conclusions

Our study reported that there is no significant relation between preeclampsia and IL6RA SNP rs2229238. Also, both the case and the control groups did not differ significantly in allele frequencies. More research is required to investigate the association between IL-6 gene polymorphism and preeclampsia risk at different gestational ages and also different populations.
